# Antisickling and toxicological evaluation of the leaves of *Scoparia dulcis* Linn (Scrophulariaceae)

**DOI:** 10.1186/s12906-015-0928-5

**Published:** 2015-11-23

**Authors:** Tavs A. Abere, Chiagozie J. Okoye, Freddy O. Agoreyo, Gerald I. Eze, Rose I. Jesuorobo, Clement O. Egharevba, Pauline O. Aimator

**Affiliations:** Department of Pharmacognosy, Faculty of Pharmacy, University of Benin, Benin City, Nigeria; Department of Physiology, College of Medical Sciences, University of Benin, Benin City, Nigeria; Department of Anatomy, College of Medical Sciences, University of Benin, Benin City, Nigeria

**Keywords:** *Scoparia dulcis*, Scrophulariaceae, Sickle cell disorders, Toxicological

## Abstract

**Background:**

*Scoparia dulcis* Linn (Scrophulariaceae) together with other medicinal plants serve as antisickling remedies in Africa. This study was aimed at investigating the antisickling activity of the leaves of the plant as well as establishing the toxicological profile.

**Method:**

Chemical tests were employed in phytochemical investigations. Evaluation of the antisickling activity involved the inhibition of sodium metabisulphite-induced sickling of the HbSS red blood cells obtained from confirmed sickle cell patients who were not in crises. Concentrations of the crude extract and its fractions were tested with normal saline and p-hydroxybenzoic acid serving as controls. Acute toxicological evaluation was carried out in mice while 30-day assessment was done in rats.

**Results:**

Phytochemical screening revealed the presence of alkaloids, tannins, flavonoids and saponins. Percentage sickling inhibitions of the aqueous methanol extracts of *S. dulcis* were significant all through the period of assay *p* < 0. 05 compared to normal saline, but not significant with PHBA. The fractions had less activity compared to the crude extracts. The LD 50 of the extract in mice was above 8000 mg/kg body weight when administered orally. Toxicological evaluations at 250 and 500 mg/kg showed mild congestion in virtually all the target organs.

**Conclusion:**

The antisickling results confirmed traditional usage of *Scoparia dulcis* in the management of Sickle cell disorders and a candidate for further investigations.

## Background

*Scoparia dulcis* Linn belongs to the Scrophulariaceae family. Common names include goatweed [1], scoparia weed, sweet broom in English, tape cava, tapixaba, vassourintia in Portuguese and escobillo in Spanish [2]. It is also known as roma fada in Hausa and aiya in Ibo [3]. It is native to the Neotropics, but it can be found throughout the tropical and subtropical world. Ethnomedicinally, *S. dulcis* is used to manage sickle cell disease in Nigeria [4], used for anaemia, burns, headache in Nicaragua [5], used to treat diabetes in India and hypertension in Taiwan [6] and also for bronchitis [7].

The diterpenoid Scoparinal, isolated from the plant demonstrated significant analgesic and anti-inflammatory activities (*P* < 0.001) in animals [8]. Scopadulcic acid B inhibited TPA-enhanced phospholipid synthesis in cultivated cells and inhibited the effect of TPA on skin tumor formulated in mice initiated with 7, 12 dimethylbenzyl anthracene [9]. The acetylated flavones glycosides of *S. dulcis* have NGR-potentiating activity which may be useful in treating neurological disorders [10]. High Performance Liquid Chromatography (HPLC) analysis of the aqueous fraction of *S. dulcis* revealed the presence of noradrenalins and adrenaline which had sympathomimetric effects [11].

Sickle Cell Disease (SCD) is one of the most prevalent morbidity and mortality diseases in Africa [12], but it also affects persons of Mediterranean, Caribbean, South and Central American, Arab and East Indian origins [13]. In Nigeria, the most common type of SCD is the homozygous (HbSS) form i.e. SCA. Estimates show that 25 % of the Nigerian population are “carriers” of the Sickle cell trait i.e. HbAS [14]. SCD is a genetic blood disorder characterized by red blood cells (RBC) that assume an abnormal rigid sickled shape. The disease stems from inadequate oxygen transport by red blood cells. In vivo, sickled erythrocytes tend to block capillaries, causing stasis, and thereby starve organs of nutrients and oxygen, eventually causing hypofunction and complete tissue destruction [15]. Sickling decreases the RBC’s flexibility and results in risk of various complications such as chronic renal failure [16], retinopathy [17], pulmonary hypertension [18], chronic pain [19], ischemia [20], stroke [21], priapsm and infraction of the penis [22].

Management of sickle cell is aimed at relieving pain, preventing infections and management of complications. First line clinical management of sickle cell anaemia includes use of hydroxyurea, folic acid, amino acids and blood transfusion to stabilize the patient’s haemoglobin level [23]. These are quite expensive and have attendant risk factors, thereby gradually paving way for the consideration of condiments from natural sources as antisickling remedies [24]. The increasing interest in these condiments is not unconnected with the general innocuous nature of their sources, which most often are herbs and even at times food crops. Researches into antisickling properties of medicinal plants have been rewarding. This alternative therapy using phytomedicines has proven to not only reduce crises but also reverse sickling. Attempts to find alternative, cheaper, and less toxic therapies led to the scientific discovery of antisickling properties of some medicinal plants such as *Cajanus cajan* seeds, *Zanthoxylum zanthoxyloides* (*Fagara*) root, *Carica papaya* unripe fruit, and also *Parquetina nigrescens* whole plant extracts which boost blood volume – all these are locally used by traditional healers in Nigeria for diverse herbal remedies [23]. This study was designed to investigate the antisickling activity as well as the toxicological profile of *Scoparia dulcis* Linn (Euphorbiaceae), one of the recipes which have been used with acclaimed success by traditional healers in Nigeria in managing sickle cell anaemia.

## Methods

### Collection of plant

The leaves of *Scoparia dulcis* Linn (Euphorbiaceae) were collected in Benin City, Edo State, Nigeria. The plants were authenticated by the curator at the Herbarium of the Department of Pharmacognosy, Faculty of Pharmacy, University of Benin, Benin City where a voucher specimen was deposited.

### Animals

Swiss albino mice (25. 92 ± 1. 05 g) and Wistar rats (220. 00 ± 16. 44 g) of both sexes were obtained from the Animal House, Department of Pharmacology and Toxicology, Faculty of Pharmacy, University of Benin, Benin City. All the animals were kept under standard environmental conditions and were handled according to international protocol for use of animals in experiments [25]. They were fed with standard pellets and tap water *ad libitum*. Ethical approval for the study was obtained from the College of Medicine, University of Benin Animal Ethics Committee (ADM/F. 22A/Vol. viii/349).

### Phytochemical investigation

Chemical tests were employed in the preliminary phytochemical screening for various secondary metabolites such as tannins (phenazone; iron complex; formaldehyde and modified iron complex tests were carried out on the aqueous extract to detect the presence of hydrolysable, condensed and pseudo tannins); cardiac glycosides (Keller - Killiani and Kedde tests were carried out on the methanolic extract to detect the presence of a deoxy sugar and to indicate the presence of a lactone ring on the cardenolides respectively); alkaloids (Mayer’s, Dragendorff’s, Wagner’s and 1 % picric acid reagents to detect the presence of alkaloidal salts and bases), saponins glycosides (frothing of the aqueous extract when shaken and haemolysis test on blood agar plates were carried out to indicate and confirm the presence of saponins); anthracene derivatives (Borntrager’s test for combined and free anthraquinones, where aglycones were extracted using chloroform and shaken with dilute ammonia) and cyanogenetic glycosides (sodium picrate paper test were used to test for the presence of hydrocyanic acid in the sample. Conversion to sodium isopurpurate indicates the presence of cyanogenetic glycosides) [26, 27].

### Extraction and fractionation

The fresh leaves of *Scoparia dulcis* Linn were air–dried for 72 h and powdered using an electric mill. The powdered leaves (4.2 kg) were extracted with MeOH-H_2_O (50: 50) (5 × 2 L). Evaporating the solvent on a water bath yielded an extract (0.78 kg) which was subsequently re-suspended in water and successively partitioned into petroleum ether (3 × 2 L), chloroform (3 × 2 L) and n-butanol (3 × 2 L). The fractions were concentrated *in vacuo* at 40 °C and used for antisickling experiments.

### Antisickling screening

#### HbSS blood samples

HbSS Blood samples were collected by venipuncture from confirmed sickle cell patients not in crises on their clinic days at the Consultant Outpatient Department (COPD) of the University Teaching Hospital, Benin City, Nigeria. None of the patients used was recently transfused with HbAA blood.

### Antisickling activity evaluation

The evaluation of the leaf extract and fractions of *Scoparia dulcis* Linn for antisickling activities was carried out using a modified method of Moody and co-workers [28]. Venipuncture blood samples from sickle cell anaemia patients not in crises were collected into EDTA bottles. Collected samples were centrifuged to remove the serum. The resulting packed erythrocytes were washed three times with sterile normal saline and centrifuged each time to remove the supernatant. 0.5 ml of the washed erythrocytes were mixed each with 0.5 ml of the different concentrations of the aqueous methanol extract (100, 300 and 500 mg/ml) or fractions (500 mg/ml) in uncovered test tubes. A 5 mg/ml solution of ρ-hydroxybenzoic acid (PHBA) in normal saline was used as the positive control while normal saline served as negative control. Samples were taken from the different mixtures and the remaining portions of the mixtures incubated for 3 h, shaking occasionally.

A 2 % Sodium metabisulphite (0.5 ml) was added to each mixture to deoxygenate the system, mixed thoroughly and sealed with liquid paraffin. Samples were taken in five replicates from the different mixtures at 0 min and at subsequent 30 min interval until seven readings were obtained.

Each sample was smeared on a microscopic slide, fixed with 95 % methanol, dried and stained with giemsa stain. Each slide was examined under the oil immersion light microscope and counting of 100 red cells in each sample. The numbers of both sickled and unsickled red blood cells were counted and the percentage of unsickled cells determined.

### Toxicological evaluation

Swiss albino mice, divided into 6 groups of 5 animals per group were orally administered the extract at doses of 1000, 2000, 3000, 4000, 5000, 6000, 7000 and 8000 mg/kg. The control group received only the vehicle (normal saline 5 ml/kg). Each group of mice was placed in the test cage for a 30–min habituation period before drug administration. The animals were observed for 10 min for the first 6 h and 10 min each day for the next two days. Lethality and gross toxicological features (convulsion, diarrhea, hyperactivity and pile-erection) were recorded for each group [29]. The animals were further observed for fourteen days.

Thirty Wistar rats were randomly distributed into three groups of ten rats each. The first (A) group served as control and received 5 ml/kg of normal saline (vehicle) while the second (B) and third (C) groups received oral doses of 250 and 500 mg/kg per day of the extract respectively for 30 consecutive days. The animals were observed for signs of toxicity (abnormal behaviours, writhing, convulsion, mood, motor activity and general body conditions) for 30 min each day. At the end of 30 days, the rats were sacrificed under chloroform anesthesia. Livers, lungs, hearts and testis were removed and preserved in 10 % formaldehyde solution. Each organ was sectioned (6 μ thick) embedded in paraffin wax and stained with hematoxylin and eosin [30].

### Statistical analysis

Data are expressed as mean ± SEM. The differences between the means were analyzed using one way analysis of variance (ANOVA). Values of *P* < 0.05 were taken to imply statistical significance between compared data.

## Results

### Phytochemical screening

Phytochemical screening of the leaves of *S. dulcis* revealed the presence of tannins, flavonoids, saponins and alkaloids (Table 1).

**Table 1:** Phytochemical constituents of *S. dulcis* leaves

Classes of secondary metabolites	Inferences
Alkaloids	+
Tannins	+
Flavonoids	+
Anthracene derivatives	-
Saponin glycosides	+
Cardiac glycosides	-
Cyanogenetic glycosides	-

Key:

- = absent; + = present

### Sickling inhibitory activities of crude extracts and fractions of *S. dulcis*

Percentage sickling inhibition of the various doses of *S. dulcis* extracts were significant all through the period of assay (*p* < 0. 05) (Table 2). There was no significant difference between the antisickling activity exhibited at 100 and 300 mg/ml. The petroleum ether and n-butanol fractions were devoid of inhibitory activities.

**Table 2:** The sickling inhibitory activities of *Scoparia dulcis* crude extracts and fractions

Time of incubation (min)	Percentage inhibition (%)								
	A*	B**	C	D	E	F	G	H	I
0 time before incubation	59.2 ± 0.12	79.0 ± 0.22	59.8 ± 1.04	58.2 ± 0.52	60.0 ± 1.34	36.4 ± 0.68	58.0 ± 0.81	30.6 ± 0.72	57.0 ± 0.50
30	50.2 ± 0.31	76.2 ± 0.05	48.0 ± 0.08	55.2 ± 1.17	60.0 ± 0.66	29.0 ± 1.87	58.2 ± 0.86	35.2 ± 1.20	58.0 ± 1.92
60	49.0 ± 0.64	77.0 ± 1.31	54.2 ± 0.12	57.0 ± 0.82	66.2 ± 1.24	28.0 ± 0.66	59.0 ± 1.34	30.2 ± 1.35	56.0 ± 0.04
90	43.4 ± 0.27	73.0 ± 0.67	49.4 ± 1.65	55.4 ± 0.29	69.0 ± 0.95	20.2 ± 1.52	60.6 ± 1.10	31.2 ± 0.07	50.4 ± 1.66
120	43.2 ± 0.32	76.0 ± 0.04	59.0 ± 1.04	60.0 ± 1.06	75.2 ± 0.73	29.8 ± 1.34	58.0 ± 0.26	28.0 ± 1.32	45.4 ± 0.47
150	40.4 ± 0.41	69.2 ± 1.18	50.6 ± 0.06	52.0 ± 1.44	55.0 ± 1.42	28.0 ± 0.56	48.0 ± 1.05	20.0 ± 0.45	43.0 ± 1.40
180	39.0 ± 0.52	65.4 ± 1.06	50.0 ± 0.82	50.2 ± 0.85	52.4 ± 0.54	25.2 ± 1.47	45.0 ± 1.12	21.4 ± 0.73	43.0 ± 1.33

Key:

A = blood + Normal saline + Sodium metabisulphite

B = blood + PHBA + Sodium metabisulphite

C = blood + Crude extract of *S. dulcis* leaf at 100 mg/ml + Sodium metabisulphite

D = blood + Crude extract of *S. dulcis* leaf at 300 mg/ml + Sodium metabisulphite

E = blood + Crude extract of *S. dulcis* leaf at 500 mg/ml + Sodium metabisulphite

F = blood + Petroleum ether fraction + Sodium metabisulphite

G = blood + Chloroform fraction + Sodium metabisulphite

H = blood + N-butanol fraction + Sodium metabisulphite

I = blood + Aqueous fraction + Sodium metabisulphite

Percentage sickling inhibitions of the aqueous methanol extracts of *S. dulcis* were significant all through the period of assay *P* < 0. 05 compared to normal saline*, but not with PHBA**

### Toxicological evaluation

The aqueous extract of *S. dulcis* did not produce any mortality up to the oral dose level of 8 g/kg body weight in mice. There were no changes in behaviour, posture, nature and frequency of stooling, mood and motor activity. The animals did not convulse, exhibit writhing or die.

**Fig. 1:**
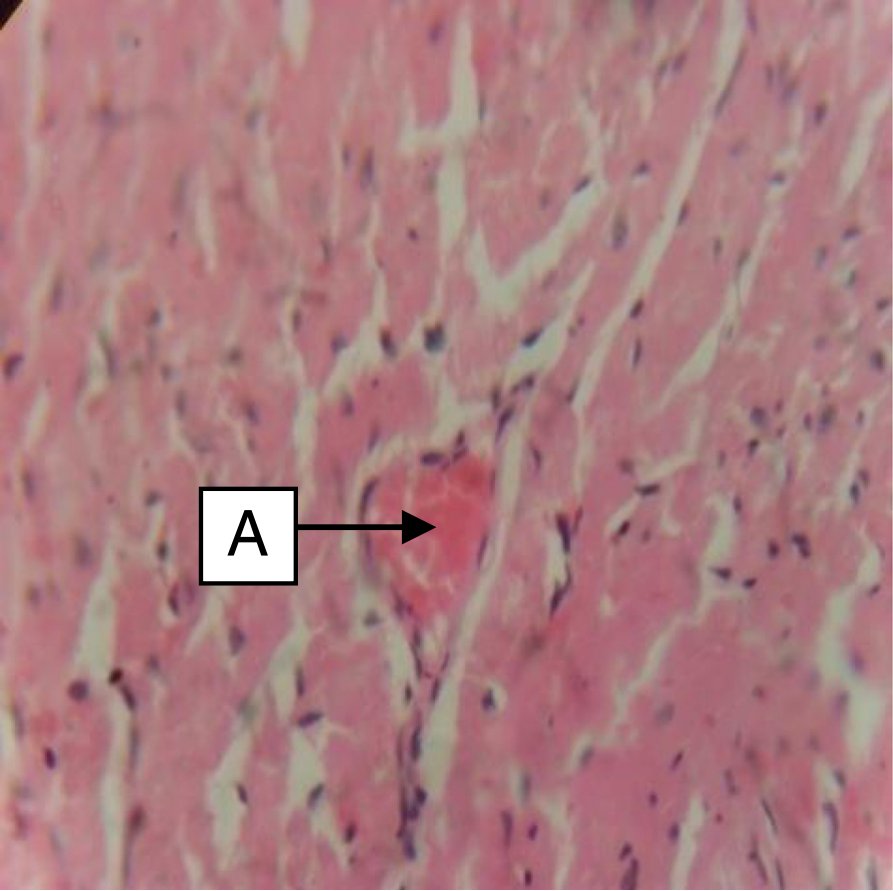
Photomicrograph of the heart of rats administered with 250 mg/kg extract of *S. dulcis* for 30 days showing mild vascular congestion A (H&E × 400)

**Fig. 2:**
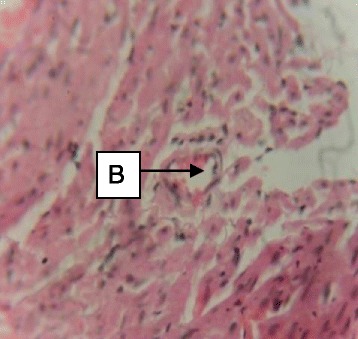
Photomicrograph of the heart of rats administered with 500 mg/kg extract of *S. dulcis* for 30 days showing mild vascular congestion B (H&E × 400)

**Fig. 3:**
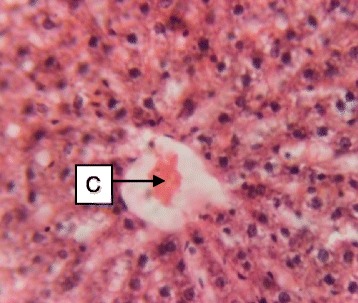
Photomicrograph of the liver of rats administered with 250 mg/kg extract of *S. dulcis* for 30 days showing mild vascular congestion C (H&E × 400)

**Fig. 4:**
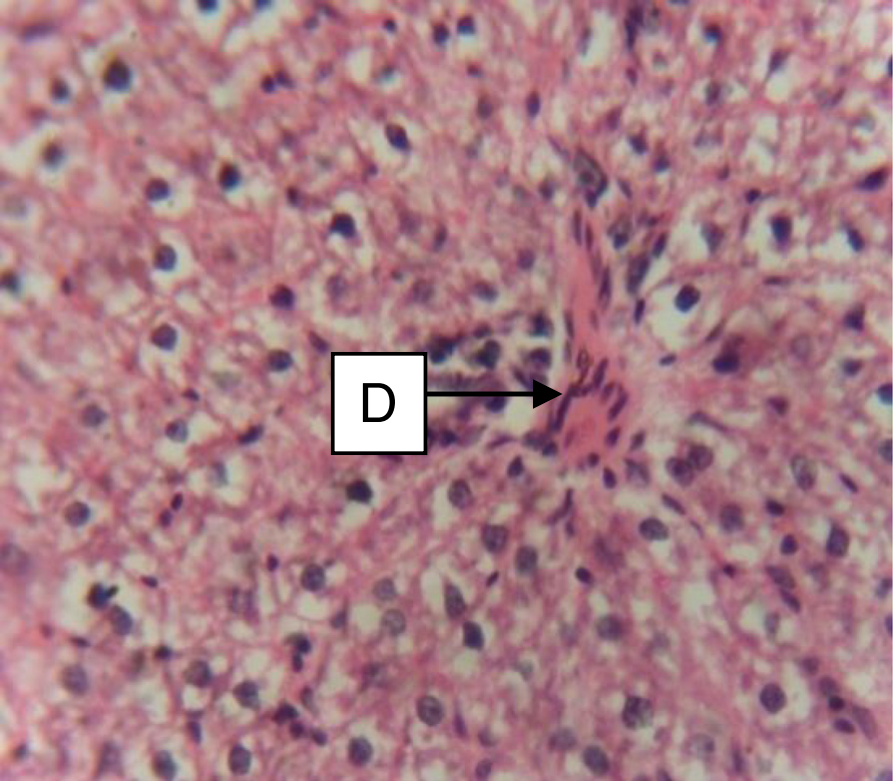
Photomicrograph of the liver of rats administered with 500 mg/kg extract of *S. dulcis* for 30 days showing mild portal congestion D (H&E × 400)

**Fig. 5:**
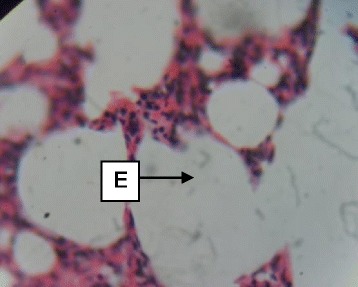
Photomicrograph of the lung of rats administered with 250 mg/kg extract of *S. dulcis* for 30 days showing unremarkable alveoli E (H&E × 400)

**Fig. 6:**
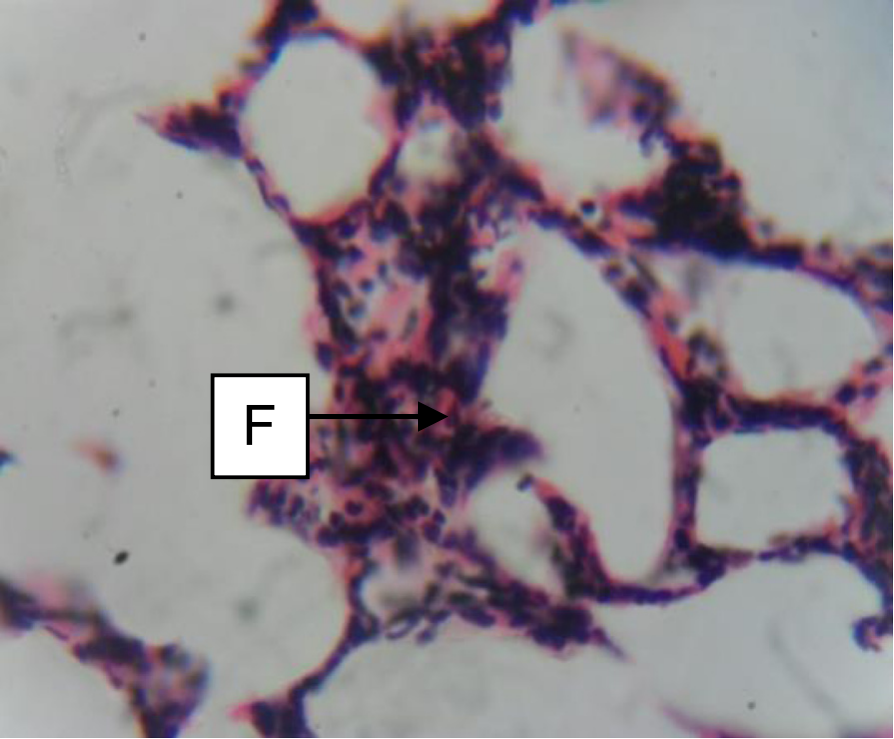
Photomicrograph of the lung of rats administered with 500 mg/kg extract of *S. dulcis* for 30 days showing mild interstitial congestion F (H&E × 400)

**Fig. 7:**
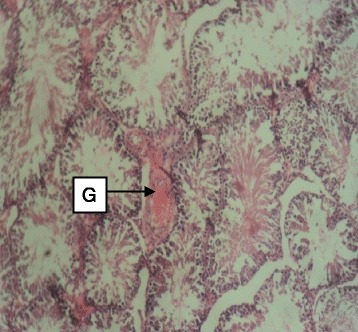
Photomicrograph of the testis of rats administered with 250 mg/kg extract of *S. dulcis* for 30 days showing moderate stroma congestion G (H&E × 400)

**Fig. 8:**
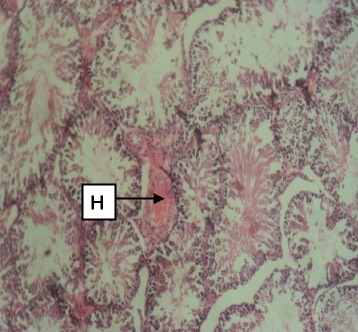
Photomicrograph of the testis of rats administered with 500 mg/kg extract of *S. dulcis* for 30 days showing moderate stroma congestion H (H&E × 400)

Daily administration of the extract for 30 days did not produce gross toxicological symptoms or deaths. Histopathology of the heart (Figs. 1 and 2) and liver (Figs. 3 and 4) showed mild vascular and portal congestions respectively. There was no degeneration of tissues in the lungs (Figs. 5 and 6) and testis (Figs. 7 and 8) at both doses except for mild interstitial congestions.

## Discussion

The Pharmacological activities of a given medicinal plant are associated with the type and nature of secondary plant metabolites present. Phytochemical evaluation of the leaves of *S. dulcis* revealed the presence of tannins, flavonoids, saponins and alkaloids. These compounds detected in *S. dulcis* are known to possess medicinal properties and health promoting effects [31]. Previous investigations have attributed the antisickling activity of many medicinal plants used in the management of sickle cell diseases to their inherent phytochemicals. Saponins and flavonoids present in *Hymenocardia acida* leaves were responsible for its antisickling activity [32]. Similarly, anthraquinones, steroidal and cardiac glycosides present in *Cissus populnea* L. contributed to the anti-sickling properties of Ajawaron, an herbal product that is marketed in Nigeria [28].

The *in-vitro* techque adopted in the antisickling efficacy bioassay was based on the simulation of the major *in-vivo* sickling-precipitating factor (i.e. reduction of oxygen tension), using sodium metabisulphite as a physiologically acceptable reducing agent. Complete sickling of the sickle cell blood sample was brought about by the use of sodium metabisulpite solution (2%w/v) which helped to suck up oxygen from the red blood cell thus created a state of reduced oxygen tension which mimics the events that occurs during sickle cell crisis. This causes the cell to assume characteristic sickle cell shapes such as the crescent, holly leaf and spindle shapes. The use of erythrocyte suspension instead of whole blood was particularly essential in ruling out the possibility of interactions of plasma component and products of their several immunological reactions and certain metabolic co-factors in general with the red blood cells [14]. Such interactions could significantly affect the shape and size of red blood cells and in the process inadvertently produce false negative or false positive results.

Aqueous and ethanol extracts of several phytomedicines have been evaluated for *in vitro* antisickling activity. Recent studies support some of the claims of traditional healers and suggest a possible correlation between the chemical composition of these plants and their uses in traditional medicine [33]. The aqueous methanol extracts of *S. dulcis* showed significant inhibitory effects at the concentrations (100, 300 and 500 mg/ml) on sodium metabisulphite-induced sickling. The chloroform and aqueous fractions of the crude extract also inhibited sodium metabisulphite induced sickling of the HbSS red blood cells to varying degrees. The inhibitory activity of *S. dulcis* could be due to the presence of bioactive compounds. The antisickling activity could be linked to the ability of the bioactive compounds present in *S. dulcis* to either inhibit *in vitro* polymerization of haemoglobin or to some structural modification linked to the environment of haemoglobin by the extracts and fractions [34].

Toxicological studies for all herbal medicines including the determination of their median lethal dose (LD 50) and other such parameters essential for a proper dosage are desirable and necessary. If there is the suspected need for more detailed studies, such herbal medicines may be subjected to sub-acute tests. The general purpose of the sub-acute toxicity tests is to determine the organs that are likely to be susceptible to toxicity by the herbal medicines. Histopathological effects of the administration of 250 and 500 mg/kg per day of the extract of *S. dulcis* to rats showed no evidence of tissue necrosis on the heart, liver, lung and testis. There were no marked adverse alterations or degeneration of tissues since these vital organs showed normal architectures suggesting no morphological disruptions as compared with the control group. It is an indication of the low toxicity of the extract [29], therefore *S. dulcis* could be said to be relatively safe.

## Conclusion

On the basis of the results obtained from the pharmacological investigations, it could be said that *Scoparia dulcis* possesses antisickling properties, indicating that it has a role in the treatment of sickle cell disorders and a good candidate for further investigations.

### Ethical approval

Ethical approval for the study was obtained from the College of Medicine, University of Benin Animal Ethics Committee (ADM/F. 22A/Vol. viii/349).

Approval was also obtained from the Sickle Cell Centre, Benin City, Nigeria through the Edo State Health Management Board for the use of consenting sickle cell patients for the study.
